# The association between chronic back problems and poor sleep quality among Ontarian adults aged 60 years and older: A cross-sectional study analyzing the Canadian Community Health Survey 2015–2016

**DOI:** 10.17269/s41997-025-01123-4

**Published:** 2025-11-23

**Authors:** Pegah Rahbar, Jessica J. Wong, Dan Wang, Efrosini Papaconstantinou, Sheilah Hogg-Johnson, Pierre Côté

**Affiliations:** 1 Department of Graduate Studies, Canadian Memorial Chiropractic College, North York, ON Canada; 2 Institute for Disability and Rehabilitation Research, Ontario Tech University, Oshawa, ON Canada; 3 Institute for Health Policy, Management, and Evaluation, University of Toronto, Toronto, ON Canada; 4 Faculty of Health Sciences, Ontario Tech University, Oshawa, ON Canada; 5 School of Physical Therapy, Faculty of Health Sciences, Western University, London, ON Canada; 6 Populations and Public Health Research Program, ICES Western, Toronto, ON Canada; 7 Department of Epidemiology and Biostatistics, Schulich School of Medicine & Dentistry, Western University, London, ON Canada; 8 Department of Research and Innovation, Canadian Memorial Chiropractic College, North York, ON Canada

**Keywords:** Older adults, Chronic back pain, Low back pain, Sleep quality, Cross-sectional, Personnes âgées, Dorsalgie chronique, Lombalgie, Qualité du sommeil, Transversal

## Abstract

**Objectives:**

Poor sleep quality’s risk factors remain understudied in aging populations. Chronic back problems (CBP), a leading cause of disability, may contribute to poor sleep quality.

Firstly, we determined the prevalence of poor sleep quality. Secondly, we investigated the association between chronic back problems and poor sleep quality, controlling for covariates. Lastly, we explored the interaction between sleep apnea and chronic back problems with respect to their association with poor sleep quality.

**Methods:**

We analyzed data from Ontarians ≥ 60 years in the 2015–2016 Canadian Community Health Survey. We estimated the prevalence (95% confidence interval (CI)) of poor sleep quality. We investigated the association between CBP and poor sleep quality to compute prevalence ratios (PR) and 95% CI, controlling for covariates. We explored the interaction between CBP and sleep apnea in their association with poor sleep quality using four combined exposure categories.

**Results:**

Overall, 52% of participants reported sleeping < 7 h or ≥ 9 h, 18% reported trouble going to/staying asleep, and 13% never/rarely felt refreshed. Controlling for covariates, CBP was associated with sleeping outside of recommended guidelines (PR = 1.19 (1.11–1.27)), trouble going to sleep or staying asleep (PR = 1.54 (1.32–1.79)), and rarely/never having refreshing sleep (PR = 1.45 (1.20–1.79)). No interaction was found between CBP and sleep apnea.

**Conclusion:**

We found that CBP is associated with poor sleep quality among Ontarians ≥ 60 years. Future studies should investigate CBP as a potential risk factor for poor sleep quality.

**Résumé**

**Objectifs:** Les facteurs de risque d’un sommeil de mauvaise qualité sont encore sous-étudiés dans les populations vieillissantes. Les problèmes de dos chroniques (PDC), l’une des principales causes d’invalidité, pourraient contribuer à la mauvaise qualité du sommeil.

En premier lieu, nous avons déterminé la prévalence de la mauvaise qualité du sommeil. En deuxième lieu, nous avons étudié l’association entre les problèmes de dos chroniques et la mauvaise qualité du sommeil après avoir apporté des ajustements pour tenir compte des effets de covariables. Pour terminer, nous avons exploré l’interaction entre l’apnée du sommeil et les problèmes de dos chroniques du point de vue de leur association avec la mauvaise qualité du sommeil.

**Méthodes:** Nous avons analysé les données d’Ontariennes et d’Ontariens de ≥ 60 ans ayant participé à l’Enquête sur la santé dans les collectivités canadiennes de 2015–2016. Nous avons estimé la prévalence (intervalle de confiance [IC] de 95%) de la mauvaise qualité du sommeil. Nous avons étudié l’association entre les PDC et la mauvaise qualité du sommeil pour calculer des ratios de prévalence (RP) et des IC de 95%, après avoir apporté des ajustements pour tenir compte des effets de covariables. Nous avons exploré l’interaction entre les PDC et l’apnée du sommeil du point de vue de leur association avec la mauvaise qualité du sommeil à l’aide de quatre catégories d’exposition combinées.

**Résultats:** Dans l’ensemble, 52% des personnes participantes ont déclaré dormir < 7 heures ou ≥ 9 heures, 18% ont dit avoir du mal à s’endormir ou à rester endormies, et 13% ont dit ne jamais ou rarement se sentir reposées. Après avoir apporté des ajustements pour tenir compte des effets de covariables, les PDC étaient associés au non-respect des directives de sommeil recommandées (RP = 1,19 [1,11–1,27]), à la difficulté de s’endormir ou de rester endormi (RP = 1,54 [1,32–1,79]) et au fait de ne jamais/rarement avoir un sommeil réparateur (RP = 1,45 [1,20–1,79]). Nous n’avons observé aucune interaction entre les PDC et l’apnée du sommeil.

**Conclusion:**

Selon nos constatations, les PDC sont associés à la mauvaise qualité du sommeil chez les Ontariennes et les Ontariens de ≥ 60 ans. De futures études devraient porter sur les PDC en tant que facteur de risque potentiel d’un sommeil de mauvaise qualité.

**Supplementary Information:**

The online version contains supplementary material available at https://doi.org/10.17269/s41997-025-01123-4.

## Introduction

Aging presents emerging challenges for the management of chronic health conditions. For example, in 2022, 47.6% of Canadian older adults (≥ 65 years) reported fair or poor sleep quality, and only 55% met the national guidelines for sleep duration (7–8 h/day) (Wang et al., 2022). Older adults may frequently experience sleep disorders such as sleep apnea, which significantly impact sleep quality (Rizzo et al., 2024; Statistics Canada, 2018). With the Canadian national prevalence of about 13% in 2017, sleep apnea is three times more common among adults 60–79 years old than those 18–59 years old (Statistics Canada, 2018). This is important because poor sleep quality is associated with premature mortality, poor general health, and prevalent chronic health conditions such as diabetes, cardiovascular disorders, depression, dementia, and obesity (Herrero Babiloni et al., 2020; Miner & Kryger, 2020; Wang et al., 2022).

Little is known about the aetiology of poor sleep quality in the aging population. One hypothesized risk factor is chronic pain (Herrero Babiloni et al., 2020) with chronic back pain highly prevalent in older adults (GBD 2021 Low Back Pain Collaborators, 2023). According to the Global Burden of Disease study, back pain is the leading cause of years lived with disability globally (GBD 2021 Low Back Pain Collaborators, 2023). In 2020, it was estimated that 619 million individuals globally experienced back pain and it is estimated that this number will grow to 843 million by 2050 in part because of aging of the population. The prevalence of back pain increases with age and the prevalence of disability related to back pain peaks between the ages of 80–85.

In a recent systematic review, low back pain was associated with sleep quality in adults (≥ 60 years old) (Rahbar, 2025). However, the studies included in the review did not control for a wide range of demographic, socioeconomic, physical and mental health-related, and lifestyle factors when investigating this association (Rahbar, 2025). Focusing on specific poor sleep quality indicators could enhance our understanding to hypothesize possible underlying mechanisms of how chronic back problems relate to poor sleep quality. Therefore, investigating the association of chronic back problems with sleep quality and its indicators is necessary to inform the design of future epidemiological and clinical research and inform clinicians and patients in understanding how chronic back problems impact sleep quality in aging adults.

The purpose of this study was to investigate the association between chronic back problems and poor sleep quality in a population-based sample of Ontario adults aged ≥ 60 years. Specifically, this study aimed to:

1. Determine the prevalence of poor sleep quality, as measured by three indicators (number of hours spent sleeping each night, frequency of trouble going to sleep or staying asleep, and frequency of refreshing sleep).
2. Investigate the association between chronic back problems and the three indicators of poor sleep quality controlling for a wide range of covariates.
3. Explore the interaction between sleep apnea and chronic back problems with respect to their association with poor sleep quality.

## Methods

### Study design

We conducted a population-based cross-sectional study analyzing the 2015–2016 Canadian Community Health Survey (CCHS) (Statistics Canada). The CCHS is a cross-sectional national representative survey of Canadians 12 years and older offered in both English and French (Statistics Canada). This study was approved by the research ethics boards at the University of Toronto (#47121), Ontario Tech University (#17981), and the Canadian Memorial Chiropractic College (#2405X03).

### Source population

The 2015–2016 CCHS involved a multistage sampling strategy using the Canada labour force survey frame for adults 18 years and older and the Canada Child Benefit for children between the ages of 12 and 17 years. The sampling frame included strata (provinces, territories, urban, rural, etc.) from which health region clusters were selected using probability sampling. Private dwellings were selected randomly from each health region, and all eligible individuals were contacted to participate from each dwelling. Each health region sample size was proportional to the square root of the estimated population of that region (Statistics Canada, 2017). Participation in the survey was voluntary. Overall, the sample included 109,659 participants from all 10 provinces and three territories, representing approximately 98% of the Canadian population aged 12 years and over at the health region level (weighted *n* = 30,590,780) (Statistics Canada, 2017). The CCHS excluded individuals living in reserves and other Indigenous settlements in the provinces, full-time members of Canadian forces, the institutionalized population, and those living in Région du Nunavik and Région des Terres-Cries-de-la-Baie-James, representing less than 3% of the Canadian population (Statistics Canada, 2017).

### Study sample

Our study sample includes 9814 participants aged 60 years and older residing in Ontario, representing a weighted sample of 2,378,212 Ontarians (Statistics Canada, 2017). Our decision to restrict to adults who are 60 years old or older was informed by the United Nations’ definition of older adults (United Nations, 2024).

### Data collection

The CCHS collected information related to sociodemographic factors, health status including self-reported health and diagnosed chronic conditions, lifestyle behaviours, and healthcare utilization in the Canadian population (Statistics Canada, 2017). The CCHS survey questions were created in consultation with health data users and a panel of health experts to confirm the questions’ content relevance. Data were collected by Statistics Canada using computer-assisted telephone interview software from January 2015 to December 2016 in four 3-month periods to minimize some of the environmental and seasonal impacts on health-related characteristics.

### Chronic low back pain

Overall, chronic conditions were described as a “health condition which is expected to last or has already lasted 6 months or more and that has been diagnosed by a health professional.” For chronic low back pain, participants were asked “Do you have back problems, excluding scoliosis, fibromyalgia, and arthritis?” (Statistics Canada, 2017). Participants could answer “yes”, “no”, “refuse to answer”, or “don’t know”.

### Sleep apnea

Self-reported sleep apnea was described as a chronic condition. Participants were asked: “Have you been told by a health professional that you have sleep apnea?” (Statistics Canada, 2017). Participants could answer “yes”, “no”, “refuse to answer”, or “don’t know”.

### Poor sleep quality

According to Nelson et al., sleep quality refers to an “individual’s self-satisfaction with all aspects of sleep”. Consistent with this definition, four indicators can be used to measure sleep quality (Nelson et al., 2022). These include:

1. Sleep efficiency: The ratio of the total time asleep over the total time spent in bed (Nelson et al., 2022).
2. Sleep latency: The time it takes a person to go from the state of wakefulness to sleep (Nelson et al., (Nelson, et al., 2022)).
3. Sleep duration: The total amount of sleep minus any arousals at night (Nelson et al., 2022).
4. Wake after sleep onset (WASO): The total amount of wake time after sleep onset until the final awakening (Nelson et al., 2022).

In CCHS (Statistics Canada, 2015–2016), sleep quality was measured with three separate questions:

1. Number of hours spent sleeping each night: “How long do you usually spend sleeping each night?” Responses ranged from less than 3 h to more than 12 h. We dichotomized responses following the National Sleep Foundation’s recommendation for sleep duration for adults 65 and older, which is 7–8 h of sleep per night (Hirshkowitz et al., 2015): “7 to less than 9 h” (meeting recommended sleep duration) versus “less than 7 h or 9 or more hours” (not meeting recommended sleep duration) (Clayborne et al., 2023). This question assesses the sleep duration per night, which is related to the sleep duration indicator of sleep quality defined by Nelson et al.
2. Frequency of trouble going to sleep or staying asleep: “How often do you have trouble going to sleep or staying asleep?”: “all the time”, “most of the time”, “sometimes”, “rarely”, and “never”. We dichotomized the responses as “all the time and most of the times” versus “sometimes, rarely, and never” (Clayborne et al., 2023; Power et al., 2005; Center for Surveillance and Applied Research, Public Health Agency of Canada, 2023; Sano et al., 2019). This question is related to sleep latency and WASO as indicators of sleep quality.
3. Frequency of refreshing sleep: “How often do you find your sleep refreshing?”: “all the time”, “most of the time”, “sometimes”, “rarely”, and “never”. We dichotomized the responses as “all the time and most of the times” versus “sometimes, rarely, and never” (Clayborne et al., 2023). This question is related to the overall self-reported satisfaction with sleep quality.

### Covariates

Informed by the literature, possible covariates included biological sex, age group, socioeconomic characteristics (including education, employment, and income), alcohol intake, body mass index (BMI), smoking, depression, anxiety, comorbidities (including heart disease, high blood pressure, and diabetes), physical activity levels, self-reported health status, living arrangement, and marital status (Bento et al., 2020; Chu et al., 2022; Ferreira et al., 2013; Leung et al., 2016; Smagula et al., 2016; Taylor et al., 2014; Wong et al., 2022). No gender information was collected in CCHS 2015–2016.

## Statistical analysis

**Fig. 1:**
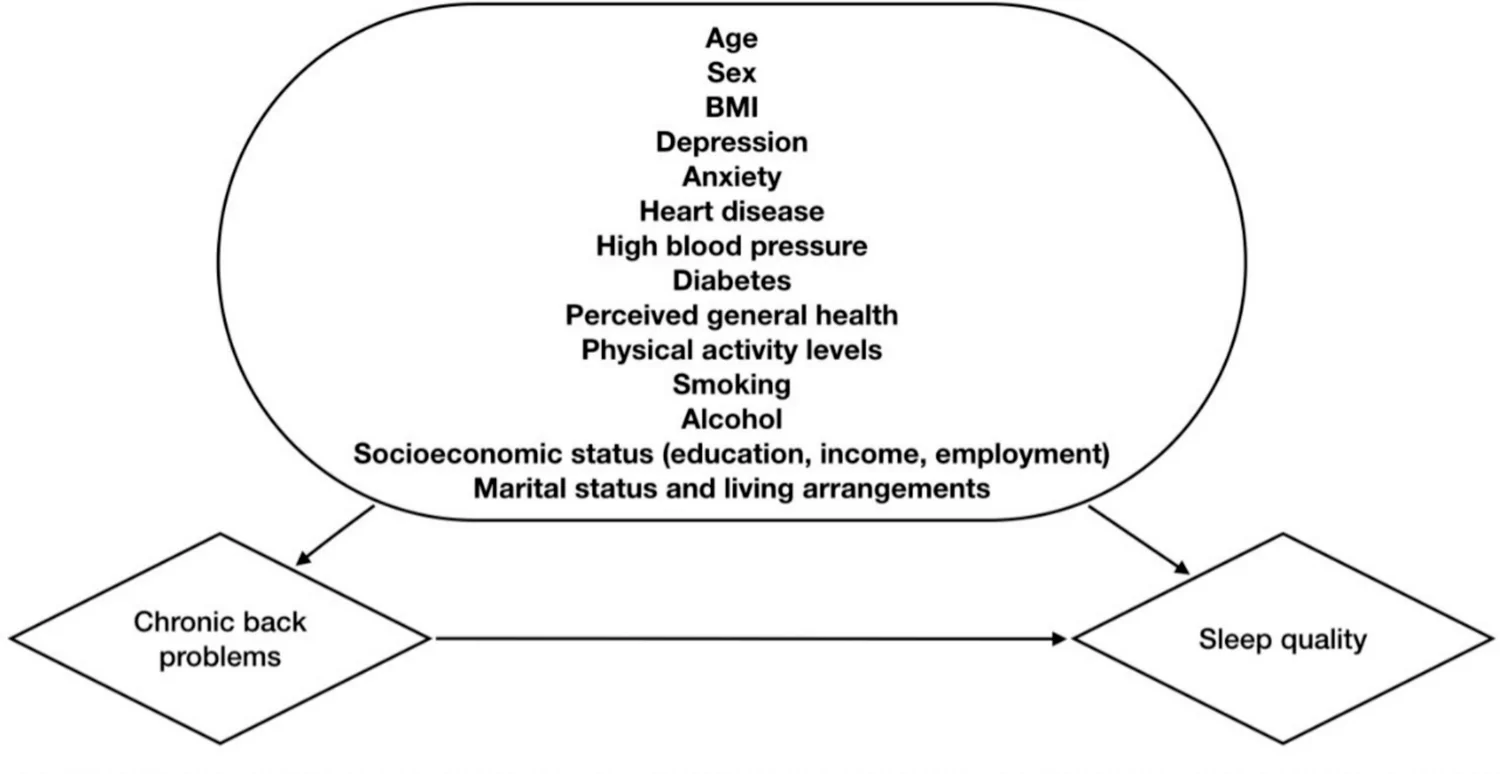
Directed acyclic graph (DAG) of the proposed association between chronic back problems and sleep quality, controlling for various covariates

We computed the prevalence and 95% confidence intervals (CI) of poor sleep quality indicators among Ontarians (≥ 60 years). Then, we built univariable and multivariable modified Poisson regression models with robust error variance (Zou, 2004) to determine the association between chronic back problems (independent variable) and each of the three indicators of sleep quality (dependent variables) while controlling for demographic, socioeconomic, lifestyle behaviour, and health-related characteristics (Fig. 1). We reported associations as prevalence ratios (PR) and 95%CI (Tamhane et al., 2016). We calculated the overall estimate as well as results stratified by sex (male, female) and age (60–69, 70–79, 80+). For all questions, we excluded participants who responded “refuse to answer” or “don’t know”, and those with a missing response from our study sample as the inclusion of such responses could potentially introduce bias to the interpretation of the study findings.

We conducted sensitivity analyses to explore the impact of potential misclassification of the outcomes when dichotomizing the responses for two sleep indicators: frequency of trouble going to sleep or staying asleep and frequency of refreshing sleep. We dichotomized the responses for both indicators as “all the time, most of the times, and sometimes” versus “rarely, and never.”

We investigated whether there is an interaction between chronic back problems and sleep apnea with respect to their association with the three indicators of poor sleep quality. We conducted modified Poisson regression analysis and included four categories for the exposure: (1) neither chronic back problems nor sleep apnea; (2) no chronic back problems and sleep apnea; (3) chronic back problems and no sleep apnea; and (4) chronic back problems and sleep apnea. We assessed whether the interactions were additive or multiplicative (VanderWeele & Knol, 2014). The additive interaction occurs when the combined effect of two exposures (i.e., chronic back problems and sleep apnea) on an outcome (i.e., sleep quality indicators) is different from the sum of their individual effects, while multiplicative interaction occurs when the two exposures’ combined effect differs from the product of their individual effects. We considered there to be no interaction between chronic back problems and sleep apnea if their calculated additive and multiplicative interactions were close to 0 and 1, respectively. We assessed whether the interactions were additive or multiplicative using the following formulas (VanderWeele & Knol, 2014):

$$\begin{array}{l}\mathrm{Additive}\;\mathrm{interaction}:\;R_{11}-R_{10}-R_{01}+1\\\mathrm{Multiplicative}\;\mathrm{interaction}:\;\frac{R_{11}}{(R_{10}R_{01)}}\end{array}$$

*R*_11_: Risk ratio when both exposures are present

*R*_10_ and *R*_01_: Risk ratio when only one of the exposures is present

The CCHS survey weights (sampling and bootstrap weights) provided by Statistics Canada were used in all analyses to produce population estimates with robust variance estimation. Sampling weights are computed to produce population-level estimates, while bootstrap weights were created to provide an appropriate estimation of the confidence intervals (Statistics Canada). We used SAS software, version [9.4] (SAS Institute Inc., Cary, NC, USA), to compute the prevalence estimates and R software version [4.4.3] (R Foundation for Statistical Computing, Vienna, Austria) to build the regression models. We used the “survey”, “boot”, and “sandwich” packages (Canty & Ripley, 2021; Lumley et al., 2024; Zeileis, 2006).

## Results

### Sample characteristics

Overall, 53.4% of the participants were female, 59.0% were 60–69 years old, 66.0% were retired, and 57.3% had a post-secondary education (Table 1). Most participants (65.6%) were married, 36.8% reported a household income of $80,000 or more, and 52.3% reported having very good to excellent general health status. Ten per cent of participants reported having sleep apnea, and 25.7% reported having chronic back problems. About 20% of participants with chronic back problems reported fair general health compared to 9.0% of participants with no chronic back problems. Similarly, among those reporting no chronic back problems, 20.1% reported excellent perceived general health as opposed to 8.8% who reported chronic back problems.

**Table 1:** Baseline characteristics of the Ontarian adults 60 and older who participated in CCHS 2015–2016 (weighted *N* = 2,378,212)

Characteristic	Categories	Total N (%)	CBP N=611,137 N (%)	No CBP N=1,767,075 N (%)
Age	60–69	1,403,729 (59.02)	368,247 (60.26)​	1,035,481(58.60)​
	70–79	706,287 (29.70)	17,8019 (29.13)​	528,269 (29.90)​
	80 and older	268,196 (10.34)	64,871 (10.61)​	203,325 (11.51)​
Sex	Female	1,269,774 (53.39)	330,811 (54.13) ​	938,963 (53.14) ​
	Male	1,108,438 (46.61)	280,326 (45.87) ​	828,112 (46.86) ​
Marital status	Married	1,559,020 (65.55)	370,881 (60.69)​	1,188,139 (67.24)​
	Common-law	102,620 (4.32)	30,183 (4.94)​	72,437 (4.10)​
	Divorced/Widowed/Separated	571,986 (24.05)	161,740 (26.47)​	410,246 (23.22)​
	Single	144,585 (6.08)	48,333 (7.91)​	96,252 (5.45)​
Main activity- Last week	Working at a paid job or business	572,088 (24.06)	128,461 (21.02) ​	443,627 (25.11)​
	Vacation (from paid work)	9489 (0.40)	2489 (0.41) ​	7000 (0.40)​
	Looking for paid work	18,522 (0.78)	7558 (1.24) ​	10,964 (0.62)​
	Going to school (including vacation from school)	5492 (0.23)	2750 (0.45) ​	2741 (0.16)​
	Caring for children	18,275 (0.77)	2255 (0.37) ​	16,020 (0.91)​
	Household work	70,515 (2.97)	20,878 (3.42) ​	49,637 (2.81) ​
	Retired	1,570,313 (66.03)	403,423 (66.01) ​	1,166,890 (66.04)​
	Long-term illness	44,941 (1.89)	26,101 (4.27)​	18,840 (1.07)​
	Volunteering	18,266 (0.77)	3281 (0.54) ​	14,985 (0.85)​
	Care-giving other than for children	5019 (0.21)	980 (0.16) ​	4039 (0.23) ​
	Other	45,291 (1.90)	12,959 (2.12) ​	32,332 (1.83)​
Living arrangement	Unattached individual living alone.	537,057 (22.58)	158,714 (25.97)​	378,344 (21.41)​
	Unattached individual living with others.	80,211 (3.37)	25,603 (4.19)​	54,608 (3.09)​
	Individual living with spouse/partner.	1,266,123 (53.24)	304,959 (49.90) ​	961,164 (54.39)​
	Parent living with spouse/partner and child(ren).	264,701 (11.13)	65,447 (10.71)​	199,254 (11.28)​
	Single parent living with children.	76,299 (3.21)	23,405 (3.83) ​	52,894 (2.99) ​
	Child living with a single parent with or without siblings.	8779 (0.37)	2606 (0.45)​	6173 (0.35)​
	Child living with two parents with or without siblings	1107 (0.05)	0 (0)​	1107 (0.06)​
	Other	143,934 (6.05)	30,403 (4.98) ​	113,531 (6.43)​
Total household income	No income or less than $20,000	171,925 (7.23)	65,497 (10.72)​	106,427 (6.02)​
	$20,000 to $39,999	489,503 (20.58)	147,277 (24.10) ​	342,227 (19.37) ​
	$40,000 to $59,999	467,818 (19.67)	125,379 (20.52)​	342,439 (19.38)​
	$60,000 to $79,999	374,320 (15.74)	95,586 (15.64)​	278,734 (15.77)​
	$80,000 or more	874,645 (36.78)	177,399 (29.03)​	697,247 (39.46)​
Highest level of education	Less than secondary	435,519 (18.31)	136,672 (22.36)​ ​	298,847 (16.91) ​
	Secondary	579,072 (24.35)	149,968 (24.54) ​	429,104 (24.28)​ ​
	Post secondary	1,363,620 (57.34)	324,497 (53.10)​ ​	1,039,124(58.80) ​
Sleep apnea	Yes	237,193 (9.97)	81,028 (13.26)	156,165 (8.84)
	No	2,141,019 (90.03)	530,108 (86.74)	1,610,910 (91.16)
Diabetes	Yes	397,099 (16.70)	124,546 (20.38)​	272,554 (15.42)​
	No	1981113 (83.30)	486,591 (79.62)​	1,494,521 (84.58)​
Heart disease	Yes	290,707 (12.22)	108,060 (17.68)​	182,647 (10.34)​
	No	2,087,505 (87.78)	503,077 (82.32)​	1,584,427 (89.66)​
High blood pressure	Yes	993,414 (41.77)	291,818 (47.75)​	701,596 (39.70)​
	No	1,384,798 (58.23)	319,319 (52.25)​	1,065,479 (60.30)​
Mood disorders	Yes	163,800 (6.89)	74,256 (12.15)​	89,544 (5.07)​
	No	2,214,412 (93.11)	536,881 (87.85)​	1,677,531 (94.93)​
Anxiety disorders	Yes	122,363 (5.15)	58,706 (9.61)​	63,657 (3.60)​
	No	2,255,849 (94.85)	552,430 (90.39)​	1,703,418 (96.40)​
Physical activity	Active	783,687 (32.95)	173,500 (28.39) ​	610,186 (34.53) ​
	Moderately active	363,722 (15.29)	89,237(14.60) ​	274,485 (15.53) ​
	Somewhat active	513,320 (21.58)	134,937(22.08) ​	378,383 (21.41) ​
	Sedentary	717,484 (30.17)	213,463 (34.93) ​	504,021 (28.52) ​
Type of smoker	Daily	223,923 (9.42)	80,443 (13.16)​	143,480 (8.12)​
	Occasionally	52,250 (2.20)	16,954 (2.77)​	35,296 (2.00)​
	Not at all	2,102,039 (88.39)	513,740 (84.06)​	1,588,299 (89.88)​
Type of alcohol drinker (past 12 months)	Regular drinker	1,366,907 (57.48)	334,102 (54.67)​	1,032,805 (58.45)​
	Occasional drinker	397,488 (16.71)	104,518 (17.10)​	292,971 (16.58)​
	Did not drink	613,817 (25.81)	172,517 (28.23)​	441,300 (24.97)​ ​
BMI classification (International)	Underweight	30,075 (1.26)	6075 (0.99)​	24000 (1.36)​
	Normal weights	916,136 (38.52)	202,860 (33.19)​	713,276 (40.36)​
	Overweight	931,578 (39.17)	236,306 (38.67)​	695,272 (39.35)​
	Obese	500,423 (21.04)	165,895 (27.15)​	334,528 (18.93)​
Perceived general health	Excellent	409,026 (17.20)	53,635 (8.78) ​	355,391 (20.11)​
	Very good	833,814 (35.06)	172,659 (28.25) ​	661,155 (37.42)​
	Good	730,865 (30.73)	192,592 (31.51) ​	538,273 (30.46)​
	Fair	282,592 (11.88)	122,981 (20.12) ​	159,612 (9.03)​
	Poor	121,914 (5.13)	69,270 (11.33) ​	52,644 (2.98)​

The percentages have been rounded up to two decimal points and therefore, might not add up to 100%

### Prevalence of poor sleep quality

In the sample overall, 51.8% (95%CI 50.0–53.6) of the participants reported sleeping outside of recommended guidelines for sleep duration (< 7 h or ≥ 9 h), 17.6% (95%CI 16.1–19.1) reported trouble going to sleep or staying asleep (all the time or most of the time), and 13.0% (95%CI 11.8–14.2) never or rarely had a refreshing sleep.

The prevalence of sleeping outside of recommended guidelines (< 7 h or ≥ 9 h) was 62.05% (95%CI 58.78–65.32) among older adults with chronic back problems compared to 48.26% (95%CI 46.18–50.34) among those without chronic back problems (Table 2). The prevalence of trouble going to sleep or staying asleep (all the time or most of the time) was 27.34% (95%CI 23.98–30.71) among those with chronic back problems compared to 14.22% (95%CI 12.59–15.84) among those without chronic back problems. Finally, the prevalence of reporting “rarely” or “never having a refreshing sleep” was 21.20% (95%CI 18.16–24.24) among those with chronic back problems compared to 10.16% (95%CI 8.98–11.35) among those without chronic back problems.

**Table 2:** Prevalence of sleep quality indicators among Ontarian adults aged 60 years and older (weighted *N* = 2,378,212)

Sleep quality indicators		CBP​ *N *= 611,137​		No CBP ​ *N* = 1,767,075​	
		***N***	Prevalence%​ (95% CI)​	***N***	Prevalence%​ (95% CI)​
Number of hours per night usually spent sleeping ​	Recommended duration ​(7 to less than 9 h)​	231,930 ​	37.95​ (34.68–41.22)​	914,284 ​	51.74​ (49.66- 53.82) ​
	Outside of duration recommendations ​	379,207 ​	62.05​ (58.78–65.32)​	852,791 ​	48.26​ (46.18–50.34)​
Frequency of trouble going to sleep or staying asleep​	Sometime, rarely, never​	444,042 ​	72.66 ​ (69.29–76.02)​	1,515,852 ​	85.78​ (84.16–87.41)​
	All the time, most of the time ​	167,095 ​	27.34​ (23.98–30.71)​	251,223 ​	14.22​ (12.59–15.84​)
Frequency of refreshing sleep ​	All the time, most of the time, sometime​	481,566 ​	78.80 ​ (75.76–81.84)​	1,587,471 ​	89.84 ​ (88.65–91.02)​
	Rarely, never ​	129,571 ​	21.20​ (18.16–24.24)​	179,604 ​	10.16 ​ (8.98–11.35)​

*CBP*, chronic back problems; *CI*, confidence interval

### Association of chronic back problems and poor sleep quality indicators

We found positive crude associations between chronic back problems and all three indicators of poor sleep quality (Table 3). After adjusting for demographic, socioeconomic, health-related, and lifestyle factors, chronic back problems remained associated with sleeping outside of recommended guidelines for sleep duration (PR = 1.19; 95%CI 1.11–1.27). Similarly, chronic back problems are associated with trouble going to sleep or staying asleep (PR = 1.54; 95%CI 1.32–1.79), and rarely or never having refreshing sleep (PR = 1.45; 95%CI 1.20–1.79). All prevalence ratios attenuated towards the null after adjusting for covariates but remained significantly higher than the reference group.

**Table 3:** Crude and adjusted prevalence ratios of chronic back problems and three poor sleep quality indicators

	Sleeping outside of recommendations per night (< 7 h or > 9 h)		Frequency of trouble going to sleep or staying asleep (all the time, most of the time)		Frequency of refreshing sleep (rarely, never)	
	cPR (95% CI)	aPR (95% CI)	cPR (95% CI)	aPR (95% CI)	cPR (95% CI)	aPR (95% CI)
No CBP	1.00 [Ref]	1.00 [Ref]	1.00 [Ref]	1.00 [Ref]	1.00 [Ref]	1.00 [Ref]
CBP	1.28 (1.20–1.38)	1.19 (1.11–1.27)	1.92 (1.63–2.27)	1.54 (1.32–1.79)	2.10 (1.73–2.51)	1.45 (1.20–1.79)

*cPR*, crude prevalence ratio; *aPR*, adjusted prevalence ratio, adjusted for sex, age group, heart disease, high blood pressure, diabetes, mood disorders, anxiety disorders, smoking, alcohol intake, body mass index category, self-reported general health, physical activity levels, marital status, living arrangements, main activity (employment), education level, and household income; *CI*, confidence interval; *CBP*, chronic back problems

Our sensitivity analysis produced similar associations, suggesting that our findings were not impacted by potential misclassification of the outcome (Appendix I). Our sex- and age-stratified analyses suggest that the association between chronic back problems and trouble going to sleep or staying asleep may be higher among males (adjusted PR = 1.92; 95%CI 1.50–2.46) and adults 80 years and older (adjusted PR = 1.91; 95%CI 1.37–2.67) (Table 4).

**Table 4:** Sex- and age-stratified analyses of the association between chronic back problems and poor sleep quality indicators among Ontarian adults 60 years and older

	Sleeping outside of recommendations per night (< 7 h or > 9 h)		Frequency of trouble going to sleep or staying asleep (all the time, most of the time)		Frequency of refreshing sleep (rarely, never)	
	cPR (95% CI)	aPR (95% CI)	cPR (95% CI)	aPR (95% CI)	cPR (95% CI)	aPR (95% CI)
Sex-stratified analysis						
No CBP in males	1.00 [Ref]	1.00 [Ref]	1.00 [Ref]	1.00 [Ref]	1.00 [Ref]	1.00 [Ref]
CBP in males	1.27 (1.15- 1.41)	1.17 (1.05–1.30)	2.51 (1.96- 3.20)	1.92 (1.50–2.46)	2.20 (1.68- 2.89)	1.50 (1.13- 2.00)
No CBP in females	1.00 [Ref]	1.00 [Ref]	1.00 [Ref]	1.00 [Ref]	1.00 [Ref]	1.00 [Ref]
CBP in females	1.29 (1.18- 1.42)	1.21 (1.10- 1.32)	1.67 (1.36–2.06)	1.36 (1.14- 1.62)	2.00 (1.54–2.60)	1.44 (1.09–1.91)
Age-stratified analysis						
No CBP in 60–69- year-olds	1.00 [Ref]	1.00 [Ref]	1.00 [Ref]	1.00 [Ref]	1.00 [Ref]	1.00 [Ref]
CBP in 60–69-year-olds	1.35 (1.23–1.49)	1.26 (1.13–1.39)	1.84 (1.45–2.34)	1.45 (1.17–1.78)	2.16 (1.69–2.77)	1.47 (1.12–1.94)
No CBP in 70–79-year-olds	1.00 [Ref]	1.00 [Ref]	1.00 [Ref]	1.00 [Ref]	1.00 [Ref]	1.00 [Ref]
CBP in 70–79-year-olds	1.17 (1.04–1.31)	1.08 (0.96–1.21)	1.96 (1.50–2.58)	1.55 (1.20–2.00)	2.04 (1.47–2.76)	1.37 (1–1.88)
No CBP in 80+-year-olds	1.00 [Ref]	1.00 [Ref]	1.00 [Ref]	1.00 [Ref]	1.00 [Ref]	1.00 [Ref]
CBP in 80+-year-old**s**	1.25 (1.08–1.45)	1.16 (1–1.36)	2.29 (1.62–3.25)	1.91 (1.37–2.67)	1.86 (1.17–2.94)	1.53 (0.95–2.47)

*cPR*, crude prevalence ratio; *aPR*, adjusted prevalence ratio, adjusted heart disease, high blood pressure, diabetes, mood disorders, anxiety disorders, smoking, alcohol intake, body mass index category, self-reported general health, physical activity levels, marital status, living arrangements, main activity (employment), education level, and household income; *CI*, confidence interval; *CBP*, chronic back problems

### Interaction between chronic back problems and sleep apnea with respect to their association with poor sleep quality indicators

We did not identify an additive or multiplicative interaction between chronic back problems and sleep apnea with respect to their association with poor sleep quality (Table 5.) (VanderWeele & Knol, 2014). The combined effect of chronic back problems and sleep apnea in their association with the three poor sleep quality indicators did not differ from the sum or product of the individual effects of chronic back problems and sleep apnea, as the additive and multiplicative interactions were close to 0 and 1, respectively (Table 5.).

**Table 5.** The results of investigating the interaction between sleep apnea and chronic back problems in their association with poor sleep quality indicators

	Sleeping outside of recommendations per night (< 7 h or > 9 h)		Frequency of trouble going to sleep or staying asleep (all the time, most of the time)		Frequency of refreshing sleep (rarely, never)	
	cPR (95% CI)	aPR (95% CI)	cPR (95% CI)	aPR (95% CI)	cPR (95% CI)	aPR (95% CI)
No CBP, no sleep apnea	1.00 [Ref]	1.00 [Ref]	1.00 [Ref]	1.00 [Ref]	1.00 [Ref]	1.00 [Ref]
No CBP, sleep apnea	1.18 (1.01–1.37)	1.15 (1.00–1.34)	1.36 (0.88–2.10)	1.37 (0.88–2.13)	1.68 (1.20–2.35)	1.48 (1.07–2.06)
CBP, no sleep apnea	1.29 (1.20–1.39)	1.21 (1.12–1.30)	1.91 (1.59–2.31)	1.55 (1.32–1.83)	2.08 (1.69–2.57)	1.50 (1.20–1.88)
CBP, sleep apnea	1.39 (1.18–1.63)	1.23 (1.05–1.44)	2.45 (1.88–3.19)	1.90 (1.44–2.50)	3.05 (2.24–4.16)	1.72 (1.34–2.42)
Interaction* (95% CI)	A: −0.13 (−0.40 to 0.14) M: 0.88 (0.70–1.11)		A: −0.02 (−0.86 to 0.82) M: 0.90 (0.52–1.55)		A: −0.26 (−1.04 to 0.52) M: 0.78 (0.47–1.27)	

*cPR,* Crude Prevalence Ratio; *aPR,* Adjusted Prevalence Ratio, adjusted for sex, age group, heart disease, high blood pressure, diabetes, mood disorders, anxiety disorders, smoking, alcohol intake, body mass index category, self-reported general health, physical activity levels, marital status, living arrangements, main activity (employment), education level, and household income; *CI*,Confidence interval; *CBP*, Chronic back problems; *SA*, Sleep apnea; *A*, Additive interaction; *M*, Multiplicative interaction

*Calculated using the results from the multivariable model

## Discussion

Our findings suggest that chronic back problems are positively associated with three indicators of poor sleep quality in Ontarian adults 60 years and older. We found that the magnitude of associations may be higher for one outcome (having trouble going to sleep or staying asleep) among males and individuals 80 years and older.

Our findings generally align with previous studies examining the association between chronic back problems and poor sleep quality, as measured by the Pittsburgh Sleep Quality Index (PSQI) (Li et al., 2010; Morelhão et al., 2021). However, few previously published studies controlled for a comprehensive set of covariates (Rahbar, 2025). Moreover, previous studies have not explored the association between chronic back problems and specific indicators of poor sleep quality, such as sleep duration, difficulty going to sleep or staying asleep, and having refreshing sleep. Focusing on specific indicators of poor sleep quality helps us refine the aetiological hypotheses to further explore the association between chronic back problems and poor sleep quality. The magnitude of the association we observed between chronic back problems and sleeping outside of the duration recommendations was consistent with findings from previous studies of poor sleep quality; however, we identified a notably stronger association for having trouble going to sleep or staying asleep and not having refreshing sleep, suggesting a potentially greater impact of chronic back problems on these two indicators of poor sleep quality than previously reported (Blay et al., 2007; Li et al., 2010; Morelhão et al., 2021). Finally, our sex- and age-stratified analysis leads to the hypothesis that chronic back problems could have a greater impact on the sleep quality of males and individuals 80 and older.

There are several biopsychosocial explanations as to why chronic back problems are associated with poor sleep quality in older adults. Although chronic pain and poor sleep quality are risk factors for each other, the possible mechanisms behind these relationships are often debated (Alsaadi et al., 2014; Herrero Babiloni et al., 2020). Various theories can help generate hypotheses about possible aetiological mechanisms for the relationship between chronic pain and sleep. Some theories describe a neurophysiological approach involving the endogenous pain modulation pathways, inflammatory markers, and hormones (e.g., dopamine and melatonin), while other theories explain the role of behavioural and psychological mechanisms involving pain catastrophizing, negative affect, and mood disorders (i.e., depression and anxiety) on poor sleep quality (Herrero Babiloni et al., 2020).

To our knowledge, this is the first population-based study to investigate the interaction between chronic back problems and sleep apnea with regard to their association with poor sleep quality. Our cross-sectional analysis does not support the hypothesis that chronic back problems and sleep apnea interact on these three indicators of poor sleep quality.

### Strengths and limitations

Our study has several strengths. First, we conducted a secondary analysis of the CCHS 2015–2016, which included a large representative sample of Ontarians and used standardized data collection methods. Second, we controlled for covariates selected a priori in our regression models built to measure the associations between chronic back problems and sleep quality. Finally, we conducted age- and sex-stratified analyses to understand how this association differed across age groups and sexes.

Our study also has limitations. First, we acknowledge that environmental and seasonal factors could potentially impact chronic back problems and poor sleep quality, but we were unable to adjust for these factors in our analysis. However, CCHS has collected data across four periods to minimize some of the environmental and seasonal impacts on health-related characteristics. Second, the chronic back problems question used in CCHS may introduce measurement error in identifying back pain as it does not explicitly describe the exact location of back problems. However, using this question provided prevalence estimates that are comparable to the global prevalence of chronic low back pain (Hoy et al., 2012). Additionally, we do not know the validity and reliability of the CCHS sleep-related questions, which may introduce measurement error related to sleep quality among older adults.

### Implications and future directions

Our cross-sectional analysis does not inform causality between chronic back problems and sleep quality. Therefore, future cohort studies are needed to determine whether chronic back problems are a risk factor for poor sleep quality among adults 60 years and older. Our study highlights the importance of considering sleep health in the comprehensive assessment and management of chronic back problems among adults 60 years and older. Clinicians may be mindful of the association of chronic back problems with sleep quality and consider strategies to assess and address poor sleep quality as part of a comprehensive approach to caring for older adults with chronic back problems. Additionally, our findings may inform future policies in healthcare to consider integrating sleep health assessment and management for older adults experiencing chronic back problems and comorbid sleep problems. Considering sleep health in older adults is important, as poor sleep quality is associated with other comorbidities and overall mortality.

## Conclusion

We found that chronic back problems are associated with three indicators of poor sleep quality among Ontarian adults 60 years and older. We also found that males and individuals 80 years and older with chronic back problems more commonly experience trouble going to sleep or staying asleep compared to those without chronic back problems. Finally, we did not find an interaction between chronic back problems and sleep apnea with regard to their association with poor sleep quality. Future cohort studies should investigate whether chronic back problems are a risk factor for poor sleep quality.

## Contribution to knowledge

What does this study add to existing knowledge?

- Chronic back problems are positively associated with sleeping outside of the sleep duration recommendations, having trouble going to sleep or staying asleep, and not having a refreshing sleep among Ontarian adults ≥ 60 years.
- The magnitude of the association between chronic back problems and having trouble going to sleep or staying asleep may be higher among males and adults 80 years and older.

What are the key implications for public health interventions, practice, or policy?

- Future cohort studies should investigate if chronic back problems are a risk factor of poor sleep quality among older adults.
- Clinicians may be mindful of sleep health in the assessment and management of older adults who experience chronic back pain
- Health policies should support integrated care models that address sleep health in older adults. This may include promoting screening for poor sleep quality in older adults with chronic back problems, and training initiatives to enhance healthcare providers’ competencies in sleep health.

## Supplementary Information

Below is the link to the electronic supplementary material.ESM 1(DOCX 15.3 KB)

## Data Availability

The data that support the findings of this study are openly available at https://odesi.ca/en/details?id=/odesi/doi__10-5683_SP3_YJBSEJ.xml

## Code availability

Data analysis codes are available upon request to the corresponding author.
