# Supplementary material for: The association between chronic back problems and poor sleep quality among Ontarian adults aged 60 years and older: A cross-sectional study analyzing the Canadian Community Health Survey 2015–2016
Source: Can J Public Health. 2025 Nov 23;117(4):658–69. doi: 10.17269/s41997-025-01123-4 (PMC13534368; doi:10.17269/s41997-025-01123-4)
Supplement: Supplementary file 1 [file 41997_2025_1123_MOESM1_ESM.docx]

**Appendix I**

**Table 1**. Sensitivity analysis results showcasing the crude and adjusted prevalence ratios of reporting chronic back problems and three poor sleep quality indicators.

|  | Frequency of trouble falling asleep or staying asleep  (All the time, most of the time, sometimes) | | Frequency of refreshing sleep  (sometimes, rarely, never) | |
| --- | --- | --- | --- | --- |
|  | **cPR (95% CI)** | **aPR (95% CI)** | **cPR (95% CI)** | **aPR (95% CI)** |
| No CBP | 1.00 [Ref] | 1.00 [Ref] | 1.00 [Ref] | 1.00 [Ref] |
| CBP | 1.38 (1.27-1.49) | 1.25 (1.15-1.35) | 1.71 (1.54-1.90) | 1.41 (1.26-1.57) |

^CBP: Chronic back problems^

^cPR: Crude prevalence ratio^

^aPR : Adjusted prevalence ratio, adjusted for sex, age group, heart disease, high blood pressure, diabetes, mood disorders, anxiety disorders, smoking, alcohol intake, body mass index category, self-reported general health, physical activity levels, marital status, living arrangements, main activity (employment), education level, and household income.^

^CI: Confidence intervals^
